# Quantifying microbial interactions based on compositional data using an iterative approach for solving generalized Lotka-Volterra equations

**DOI:** 10.1371/journal.pcbi.1013691

**Published:** 2025-11-07

**Authors:** Yue Huang, Tianqi Tang, Xiaowu Dai, Fengzhu Sun

**Affiliations:** 1 Department of Quantitative and Computational Biology, University of Southern California, Los Angeles, California, United States of America; 2 Department of Neurology, University of California, San Francisco, California, United States of America; 3 Departments of Statistics and Data Science, and Biostatistics, University of California, Los Angeles, California, United States of America; Fudan University, CHINA

## Abstract

Understanding microbial interactions is fundamental for exploring population dynamics, particularly in microbial communities where interactions affect stability and host health. Generalized Lotka-Volterra (gLV) models have been widely used to investigate system dynamics but depend on absolute abundance data, which are often unavailable in microbiome studies. To address this limitation, we introduce an iterative Lotka-Volterra (iLV) model, a novel framework tailored for compositional data that leverages relative abundances and iterative refinements for parameter estimation. The iLV model features two key innovations: an adaptation of the gLV framework to compositional constraints and an iterative optimization strategy combining linear approximations with nonlinear refinements to enhance parameter estimation accuracy. Using simulations and real-world datasets, we demonstrate that iLV surpasses existing methodologies, such as the compositional LV (cLV) and the generalized LV (gLV) model, in recovering interaction coefficients and predicting species trajectories under varying noise levels and temporal resolutions. Applications to the lynx-hare predator-prey, *Stylonychia pustula*-*P. caudatum* mixed culture, and cheese microbial systems revealed consistency between predicted and observed relative abundances showcasing its accuracy and robustness. In summary, the iLV model bridges theoretical gLV models and practical compositional data analysis, offering a robust framework to infer microbial interactions and predict community dynamics using relative abundance data, with significant potential for advancing microbial research.

## Author summary

Microbes and animals often live in communities where species interact in complex ways. Understanding these interactions is essential for predicting population dynamics and how ecosystems respond to environmental changes. A widely used mathematical tool for modeling these interactions is the generalized Lotka-Volterra model. However, it requires information on absolute population sizes, which is rarely available in microbiome studies. We developed the iterative Lotka-Volterra (iLV) model to overcome this challenge. iLV adapts the classical framework to work directly with relative abundance data—the kind most often collected in microbiome studies. Through an iterative optimization process, iLV improves the accuracy of inferred species interactions and population trajectories. We validated our approach using simulated data, along with real-world systems including the snowshoe hare–Canadian lynx system, a microbial co-culture experiment, and a cheese microbial community. In each case, iLV provided more accurate results than existing methods. Our work offers a practical framework for studying species interactions using relative abundance data.

## Introduction

Quantifying microbial interactions is essential for understanding the dynamics of microbial communities. This task has gained increased attention in microbiome research, as microbial interactions directly impact community stability and host health [1–3]. The Lotka-Volterra (LV) modeling framework was originally developed to describe predator-prey systems [4], based on which the generalized Lotka-Volterra (gLV) was proposed to model systems of more than two species. gLV has been widely adopted to infer these interactions due to its versatility in modeling nonlinear dynamics across diverse ecosystems, from microbial communities to macroscopic populations [5–7]. The generalized Lotka-Volterra (gLV) model remains a key tool for describing microbial interactions and forecasting community trajectories. Applications range from predicting microbiome responses to antibiotics and dietary shifts to understanding microbial coexistence in ecological contexts [8,9].

However, the widespread use of sequencing technologies in microbial studies introduces a unique challenge: most data are compositional, providing relative rather than absolute abundance profiles [10,11]. This poses significant limitations for traditional gLV models, which rely on absolute abundance inputs for accurate parameterization and prediction [12,13]. To address this problem, compositional adaptations of gLV models have been developed, such as the compositional Lotka-Volterra (cLV) framework, which maps dynamics onto a constrained simplex, accommodating the summation constraint of relative abundances [13].

These previous advancements have highlighted the potential of compositional data analysis, but they also have some major limitations, such as moderate accuracy due to their nature of linear approximations. For cLV, the coefficients in the gLV model cannot be fully recovered [13], making it harder to interpret the results. Gaps remain in integrating theoretical and empirical approaches for compositional data. To bridge the gap, we proposed a novel method (called iterative Lotka-Volterra or iLV) of compositional data analysis based on the traditional gLV model, emphasizing the compositional constraints and their implications for model parameterization. There are two major innovative components of iLV, including defining the classical generalized Lotka-Volterra model with relative abundances and inter-species sum of absolute abundances, and iterative linear approximations followed by non-linear optimizations. By leveraging computational simulations, we compared the performance of iLV with other existing methods including generalized Lotka–Volterra (gLV) and compositional Lotka-Volterra (cLV). We also applied iLV in three real-world datasets to estimate the coefficients of gLV model and accurately recover community relative abundance trajectories.

## Results

### Performance and stability of different non-linear optimization methods

**Fig 1:**
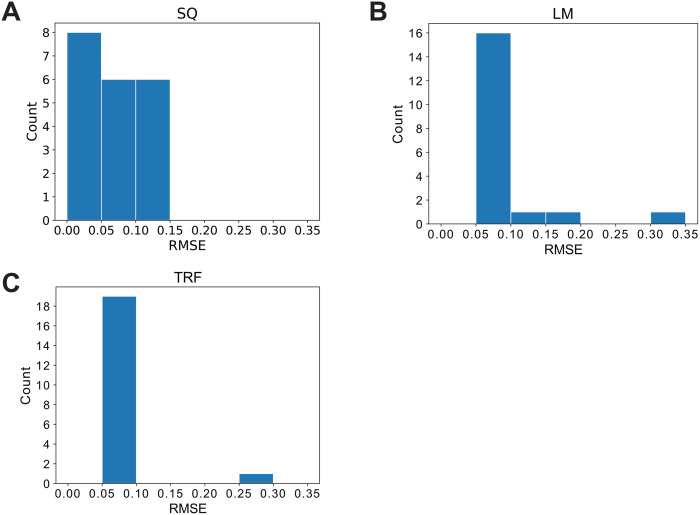
Trajectory Root Mean Square Error (RMSE) distributions of three optimization methods: (A) SQ refers to leastsq(), (B) LM refers to least_squares(method = ‘lm’), and (C) TRF refers to least_squares(method=’trf’). Data points were simulated using the following parameter setting that reflects periodic oscillations among three species: ${𝐫}_{\text{1}}=\text{0}.\text{3}\text{1}$, ${𝐫}_{\text{2}}=\text{0}.\text{6}$, ${𝐫}_{\text{3}}=\text{0}.\text{2}\text{9}$, ${𝐛}_{\text{1}\text{2}}=-\text{0}.\text{0}\text{1}$, ${𝐛}_{\text{1}\text{3}}=\text{0}.\text{011}$, ${𝐛}_{\text{2}\text{1}}=\text{0}.\text{009}$, ${𝐛}_{\text{2}\text{3}}=-\text{0}.\text{01}$, ${𝐛}_{\text{3}\text{1}}=-\text{0}.\text{012}$, ${𝐛}_{\text{3}\text{2}}=\text{0}.\text{015}$, ${𝐱}_{\text{1}}(\text{0})=\text{0}.\text{3}$, ${𝐱}_{\text{2}}(\text{0})=\text{0}.\text{5}$, ${𝐱}_{\text{3}}(\text{0})=\text{0}.\text{2}$, ${𝐍}_{sum}(\text{0})=\text{1}\text{0}\text{0}$, with a time length of 20 and a time interval of 0.4. All three methods exhibited instabilities for this dataset, while leastsq() achieved the lowest trajectory RMSE of ${9.29\times 10}^{-\text{9}}$. The mean RMSEs across 20 runs for leastsq(), least_squares(method = ’lm’), and least_squares(method = ’trf’) were 0.0687, 0.135, and 0.103, respectively, while their corresponding median RMSEs were 0.0386, 0.0958, and 0.0945.

When applying the iLV algorithm to parameter estimation, the choice of non-linear optimization methods in Subroutine 2 can influence both accuracy and numerical stability, particularly when the problem is ill-conditioned. Fig 1 presents an example where instability occurred for different non-linear optimization methods. We simulated data points using the following parameter setting that reflects periodic oscillations among three species: $r_{\text{1}}=\text{0}.\text{3}\text{1}$, $r_{\text{2}}=\text{0}.\text{6}$, $r_{\text{3}}=\text{0}.\text{2}\text{9}$, $b_{\text{1}\text{2}}=-\text{0}.\text{0}\text{1}$, $b_{\text{1}\text{3}}=\text{0}.\text{011}$, $b_{\text{2}\text{1}}=\text{0}.\text{009}$, $b_{\text{2}\text{3}}=-\text{0}.\text{01}$, $b_{\text{3}\text{1}}=-\text{0}.\text{012}$, $b_{\text{3}\text{2}}=\text{0}.\text{015}$, $x_{\text{1}}(\text{0})=\text{0}.\text{3}$, $x_{\text{2}}(\text{0})=\text{0}.\text{5}$, $x_{\text{3}}(\text{0})=\text{0}.\text{2}$, $N_{sum}(\text{0})=\text{1}\text{0}\text{0}$, with a time length of 20 and a time interval of 0.4. We executed the iLV Algorithm 20 times using the simulated data and estimated the parameters with three different non-linear optimization methods: leastsq(), least_squares(method = ‘lm’), and least_squares(method=’trf’). $N_{sum\_initial\_guess}$ was set to 200. As shown in Fig 1, all three methods exhibited instabilities potentially due to rounding errors and the ill-conditioned nature of this dataset, and leastsq() achieved the lowest trajectory RMSE of ${\text{9}.\text{2}\text{9}\times \text{1}\text{0}}^{-\text{9}}$. Therefore, to mitigate the effects of numerical instabilities and the variable performance of optimization methods across different datasets, we compared the trajectory RMSE returned by least_squares (method = “trf” or method = “lm”) and leastsq() in Subroutine 2, and retained the results with the lowest RMSE. Additionally, during benchmarking, we repeated the algorithm 20 times and reported the parameter that yielded the lowest RMSE among these 20 runs.

### Two subroutines of the iLV Algorithm jointly improve parameter estimation

**Fig 2:**
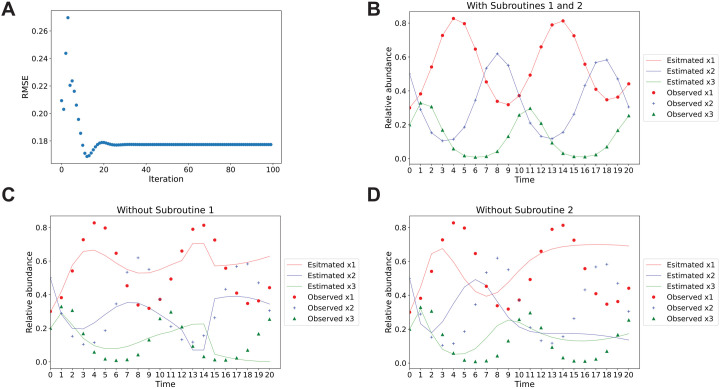
Two subroutines of the iLV Algorithm jointly improve parameter estimation. Panel **A** depicts the trajectory RMSE values across 100 iterations of the iterative subroutine in the iLV Algorithm, illustrating its ability to iteratively identify parameter estimations with the lowest RMSE within the first 100 runs. The last three panels compare the predicted relative abundances (solid lines) to observed values (symbols) for each species, with both Subroutines (Panel **B**), without Subroutine 1 (Panel **C**), and without Subroutine 2 (Panel **D**), respectively, demonstrating significantly improved alignment after joint effects of both Subroutines in the iLV Algorithm.

An accurate initial guess of parameters is crucial for the success of optimization functions like leastsq() or least_squares() in finding an optimal local minimum of the cost function. Subroutine 1 (the iterative subroutine) of the iLV Algorithm provides an iterative approach to generate such initial guesses effectively. To illustrate the impact of the iterative subroutine, we generated simulated data using the following parameter settings: $r_{\text{1}}=\text{0}.\text{3}\text{1}$, $r_{\text{2}}=-\text{0}.\text{6}$, $r_{\text{3}}=\text{0}.\text{2}\text{9}$, $b_{\text{1}\text{2}}=-\text{0}.\text{0}\text{1}$, $b_{\text{1}\text{3}}=\text{0}.\text{011}$, $b_{\text{2}\text{1}}=\text{0}.\text{009}$, $b_{\text{2}\text{3}}=-\text{0}.\text{01}$, $b_{\text{3}\text{1}}=-\text{0}.\text{012}$, $b_{\text{3}\text{2}}=\text{0}.\text{015}$, $x_{\text{1}}(\text{0})=\text{0}.\text{3}$, $x_{\text{2}}(\text{0})=\text{0}.\text{5}$, $x_{\text{3}}(\text{0})=\text{0}.\text{2}$, $N_{sum}(\text{0})=\text{1}\text{0}\text{0}$, time range = 20, time interval = 1. $N_{sum\_initial\_guess}$ was set to 200. Fig 2A shows the trajectory RMSE as a function of the number of iterations in Subroutine 1 (the iterative subroutine). The figure indicates that Subroutine 1 iteratively refined the initial guess, with the optimal guess achieved in the 13th iteration out of 100. Fig 2B and 2C illustrate how Subroutine 1 substantially improved the fit between predicted and observed relative abundances. Without applying Subroutine 1, using the parameter estimations derived from the gLV linear approximation as the starting point of Subroutine 2 (the least square estimation subroutine) resulted in poor optimization performance, with an RMSE of 0.122 for leastsq() (Fig 2C). In contrast, using the parameters from the iteration with the lowest trajectory RMSE within the first 100 iterations significantly improved optimization performance, yielding an RMSE of ${\text{2}.\text{1}\text{1}\times \text{1}\text{0}}^{-\text{1}\text{0}}$ with leastsq() (Fig 2B). Under this parameter setting, least_squares(method = ‘lm’ or ‘trf’) did not perform as well as leastsq(). Notably, the iterative subroutine guarantees non-increasing trajectory RMSE values, as the first iteration corresponds to the result without applying the iterative subroutine, and only the iteration with the lowest trajectory RMSE (e.g., out of the first 100 iterations) is selected as the starting point of the least square estimation subroutine.

Subroutine 2 helps to improve parameter estimation as leastsq() and least_squares() functions find a local minimum of the cost function F(x), near the initial guess returned by Subroutine 1. As shown in Fig 2D, the trajectory RMSE was 0.169 when only Subroutine 1 was applied without Subroutine 2.

### Benchmarking with existing methods

Previously, Joseph et al. [13] developed a nonlinear dynamical system for modeling microbial dynamics using relative abundances, termed the “compositional” Lotka-Volterra (cLV) model, which unifies methodologies based on generalized Lotka-Volterra (gLV) equations with approaches from compositional data analysis. However, cLV cannot fully recover the original interaction coefficients in the generalized Lotka-Volterra model and it also assumes $N_{sum}$ stays roughly constant [13]. There could also be other ways of dealing with relative abundance input, such as applying the generalized Lotka–Volterra (gLV) to relative abundance input directly (we referred to it as gLV_relative). Besides, we used Pearson or Spearman correlation coefficients between the relative abundance data of two microbial species i and j to assess the corresponding interaction coefficients $b_{ij}$ of the generalized Lotka–Volterra model, as two other benchmarked methods. We also applied the gLV model to absolute abundance data for parameter estimation as a baseline for comparison.

To evaluate the performance of these methods, we generated simulated data using the generalized Lotka–Volterra model, represented by the ODE system (4), under two biologically relevant parameter settings without self-interactions. The first parameter setting ($r_{\text{1}}=\text{0}.\text{3}\text{1}$, $r_{\text{2}}=-\text{0}.\text{6}$, $r_{\text{3}}=\text{0}.\text{2}\text{9}$, $b_{\text{1}\text{1}}=\;b_{\text{2}\text{2}}=b_{\text{3}\text{3}}=\;$0, $b_{\text{1}\text{2}}=-\text{0}.\text{0}\text{1}$, $b_{\text{1}\text{3}}=\text{0}.\text{011}$, $b_{\text{2}\text{1}}=\text{0}.\text{009}$, $b_{\text{2}\text{3}}=-\text{0}.\text{01}$, $b_{\text{3}\text{1}}=-\text{0}.\text{012}$, $b_{\text{3}\text{2}}=\text{0}.\text{015}$, $x_{\text{1}}(\text{0})=\text{0}.\text{3}$, $x_{\text{2}}(\text{0})=\text{0}.\text{5}$, $x_{\text{3}}(\text{0})=\text{0}.\text{2}$, $N_{sum}(\text{0})=\text{1}\text{0}\text{0}$) reflects periodic oscillations among three species, which are similar to patterns observed in the lynx-hare dynamics [14]. The second parameter setting ($r_{\text{1}}=\text{0}.\text{2}\text{1}$, $r_{\text{2}}=\text{0}.\text{4}$, $r_{\text{3}}=\text{0}.\text{1}\text{9}$, $b_{\text{1}\text{1}}=\;b_{\text{2}\text{2}}=b_{\text{3}\text{3}}=\;$0, $b_{\text{1}\text{2}}=\text{0}.\text{0}\text{2}$, $b_{\text{1}\text{3}}=\text{0}.\text{016}$, $b_{\text{2}\text{1}}=\text{0}.\text{01}$, $b_{\text{2}\text{3}}=-\text{0}.\text{014}$, $b_{\text{3}\text{1}}=-\text{0}.\text{017}$, $b_{\text{3}\text{2}}=\text{0}.\text{02}$, $x_{\text{1}}(\text{0})=\text{0}.\text{3}$, $x_{\text{2}}(\text{0})=\text{0}.\text{5}$, $x_{\text{3}}(\text{0})=\text{0}.\text{2}$, $N_{sum}(\text{0})=\text{1}\text{0}\text{0}$) describes a scenario where one species eventually dominates while others decline, as observed in the cheese microbial dataset [15].

To test robustness, we added varying levels of noise (no noise, 5%, or 10% random Gaussian noise) to the simulated data. Relative abundance data were generated for different time lengths and intervals. For each noisy group (either 5% or 10% noise), 20 randomly noisy datasets of relative abundance were generated. iLV was used to estimate the interaction coefficients with $N_{sum\_intial\_guess}=\;\text{2}\text{0}\text{0}$. The cosine similarities between the estimated and ground truth interaction coefficients ($b_{ij}$) are presented in Tables 1, 2, 4, and 5. For the cLV model, cosine similarities were calculated between the estimated and ground truth values of $b_{ij}-b_{Dj}$ (where species D has the lowest observed variance of relative abundance among all species and i ≠ j or D), due to the inherent limitations of the cLV framework of not being able to estimate $b_{ij}$ [13]. Trajectory RMSE was not used as an evaluation metric since the system can be unstable growing exponentially if parameter estimation is too far from the ground truth. Moreover, cLV cannot fully recover $b_{ij},\text{ or}$ $r_{i}$ either.

**Table 1:** Average cosine similarity of interaction coefficients (*b*_ij_) where i≠j under varying time intervals (Δt) and noise levels for different methods, using simulated datasets that reflect periodic oscillations among three species.

Time segment		iLV	cLV	gLV_relative	Pearson’s r	Spearman’s r	gLV_absolute
**No noise**	Δt = 1	**1**	0.813	0.789	0.00201	-0.00310	0.816
	Δt = 0.5	**1**	0.888	0.883	-5.23e-05	0.00791	0.945
	Δt = 0.1	**1**	0.924	0.909	-0.00283	0.000474	0.997
**Noise level 0.05**	Δt = 1	**0.936** **(0.0751)**	0.811 (0.0107)	0.784 (0.0146)	0.00213 (0.00386)	-0.00585 (0.00852)	0.810 (0.0168)
	Δt = 0.5	**0.962** **(0.0493)**	0.889 (0.0111)	0.883 (0.00896)	-0.00178 (0.00246)	0.00269 (0.00333)	0.947 (0.00888)
	Δt = 0.1	0.991 (0.0142)	0.914 (0.00524)	0.906 (0.00294)	-0.00237 (0.00143)	-5.71e-05 (0.00132)	0.995 (0.00267)
**Noise level 0.10**	Δt = 1	0.813 (0.210)	0.802 (0.0189)	0.774 (0.0273)	0.00187 (0.00684)	-0.00254 (0.0100)	0.807 (0.0465)
	Δt = 0.5	0.886 (0.109)	0.875 (0.0260)	0.866 (0.0228)	-0.00113 (0.00543)	-2.38e-4 (0.00688)	**0.937** **(0.0268)**
	Δt = 0.1	0.961 (0.0431)	0.874 (0.0177)	0.868 (0.0147)	-0.00268 (0.00218)	-1.81e-5 (0.00255)	**0.970** **(0.0226)**

For each noisy group, 20 randomly noisy datasets of relative abundance were generated, and the standard deviation was reported in parentheses following the mean cosine similarities of *b*_ij_. Best performances (Friedman Test, followed by post-hoc one-sided Wilcoxon signed-rank test, with p value less than 0.05) are highlighted in bold. The data were simulated under the first parameter setting ($r_{\text{1}}=\text{0}.\text{3}\text{1}$, $r_{\text{2}}=-\text{0}.\text{6}$, $r_{\text{3}}=\text{0}.\text{2}\text{9}$, $b_{\text{1}\text{1}}=\;b_{\text{2}\text{2}}=b_{\text{3}\text{3}}=\;$0, $b_{\text{1}\text{2}}=-\text{0}.\text{0}\text{1}$, $b_{\text{1}\text{3}}=\text{0}.\text{011}$, $b_{\text{2}\text{1}}=\text{0}.\text{009}$, $b_{\text{2}\text{3}}=-\text{0}.\text{01}$, $b_{\text{3}\text{1}}=-\text{0}.\text{012}$, $b_{\text{3}\text{2}}=\text{0}.\text{015}$, $x_{\text{1}}(\text{0})=\text{0}.\text{3}$, $x_{\text{2}}(\text{0})=\text{0}.\text{5}$, $x_{\text{3}}(\text{0})=\text{0}.\text{2}$, $N_{sum}(\text{0})=\text{1}\text{0}\text{0}$). We set $N_{sum\_intial\_guess}=\text{2}\text{0}\text{0}$.

**Table 2:** Average cosine similarity of interaction coefficients (*b*_ij_) where i≠j under varying time ranges (t) and noise levels for different methods, using simulated datasets that reflect periodic oscillations among three species.

Time range		iLV	cLV	gLV_relative	Pearson’s r	Spearman’s r	gLV_absolute
**No noise**	t = 20	**1**	0.814	0.788	-0.00325	-0.00166	0.817
	t = 15	**1**	0.843	0.822	0.0127	0.0108	0.820
	t = 10	**1**	0.813	0.789	0.00201	-0.00310	0.816
**Noise level 0.05**	t = 20	**0.980** **(0.0398)**	0.811 (0.0109)	0.784 (0.0135)	-0.00269 (0.00339)	-0.00161 (0.00366)	0.817 (0.0161)
	t = 15	**0.940** **(0.138)**	0.839 (0.0100)	0.816 (0.0116)	0.0133 (0.00265)	0.0121 (0.00437)	0.820 (0.0220)
	t = 10	**0.936** **(0.0751)**	0.811 (0.0107)	0.784 (0.0146)	0.00213 (0.00386)	-0.00585 (0.00852)	0.810 (0.0168)
**Noise level 0.10**	t = 20	**0.958** **(0.0569)**	0.813 (0.0145)	0.788 (0.0171)	-0.00587 (0.00452)	-0.00435 (0.00478)	0.824 (0.0358)
	t = 15	**0.958** **(0.0567)**	0.832 (0.0161)	0.808 (0.0216)	0.0117 (0.00557)	0.00820 (0.00786)	0.841 (0.0320)
	t = 10	0.813 (0.210)	0.802 (0.0189)	0.774 (0.0273)	0.00187 (0.00684)	-0.00254 (0.0100)	0.807 (0.0465)

For each noisy group, 20 randomly noisy datasets of relative abundance were generated, and the standard deviation was reported in parentheses following the mean cosine similarities of *b*_ij_. Best performances (Friedman Test, followed by post-hoc one-sided Wilcoxon signed-rank test, with p value less than 0.05) are highlighted in bold. The data were simulated under the first parameter setting ($r_{\text{1}}=\text{0}.\text{3}\text{1}$, $r_{\text{2}}=-\text{0}.\text{6}$, $r_{\text{3}}=\text{0}.\text{2}\text{9}$, $b_{\text{1}\text{1}}=\;b_{\text{2}\text{2}}=b_{\text{3}\text{3}}=\;$0, $b_{\text{1}\text{2}}=-\text{0}.\text{0}\text{1}$, $b_{\text{1}\text{3}}=\text{0}.\text{011}$, $b_{\text{2}\text{1}}=\text{0}.\text{009}$, $b_{\text{2}\text{3}}=-\text{0}.\text{01}$, $b_{\text{3}\text{1}}=-\text{0}.\text{012}$, $b_{\text{3}\text{2}}=\text{0}.\text{015}$, $x_{\text{1}}(\text{0})=\text{0}.\text{3}$, $x_{\text{2}}(\text{0})=\text{0}.\text{5}$, $x_{\text{3}}(\text{0})=\text{0}.\text{2}$, $N_{sum}(\text{0})=\text{1}\text{0}\text{0}$). We set $N_{sum\_intial\_guess}=\text{2}\text{0}\text{0}$.

**Table 3:** The cosine similarity of interaction coefficients (*b*_ij_) where i≠j under varying time intervals (Δt) and self-interaction levels for different methods, using simulated datasets that reflect periodic oscillations among three species.

Time segment		iLV	cLV	gLV_relative	Pearson’s r	Spearman’s r	gLV_absolute
**Low self-interaction**	Δt = 1	**0.967**	0.838	0.787	0.0613	0.0302	0.841
	Δt = 0.5	**0.966**	0.889	0.845	0.0624	0.0178	0.963
	Δt = 0.1	0.966	0.912	0.873	0.0629	0.0121	**0.998**
**Medium self-interaction**	Δt = 1	0.811	0.639	0.545	0.0843	0.102	**0.977**
	Δt = 0.5	0.815	0.683	0.574	0.0878	0.102	**0.994**
	Δt = 0.1	0.817	0.710	0.592	0.0905	0.102	**0.999**
**High self-interaction**	Δt = 1	0.754	0.432	0.365	0.0685	0.0978	**0.958**
	Δt = 0.5	0.705	0.475	0.384	0.0776	0.0998	**0.985**
	Δt = 0.1	0.715	0.503	0.398	0.0841	0.101	**0.999**

Best performances are highlighted in bold. The data were simulated under the first parameter setting ($r_{\text{1}}=\text{0}.\text{3}\text{1}$, $r_{\text{2}}=-\text{0}.\text{6}$, $r_{\text{3}}=\text{0}.\text{2}\text{9}$, $b_{\text{1}\text{1}}=-\text{0}.\text{0}\text{0}\text{1}\text{7},\;-\text{0}.\text{0}\text{0}\text{6}\text{5},\;$or −0.012 (low, medium, or high self-interactions, respectively), $b_{\text{1}\text{2}}=-\text{0}.\text{0}\text{1}$, $b_{\text{1}\text{3}}=\text{0}.\text{011}$, $b_{\text{2}\text{1}}=\text{0}.\text{009}$, $b_{\text{2}\text{2}}=-\text{0}.\text{0}\text{0}\text{2}\text{8},\;-\text{0}.\text{0}\text{0}\text{5}\text{2},\;$or −0.014 (low, medium, or high self-interactions, respectively), $b_{\text{2}\text{3}}=-\text{0}.\text{01}$, $b_{\text{3}\text{1}}=-\text{0}.\text{012}$, $b_{\text{3}\text{2}}=\text{0}.\text{015}$, $b_{\text{3}\text{3}}=-\text{0}.\text{0}\text{0}\text{4}\text{2},\;-\text{0}.\text{0}\text{0}\text{7}\text{6},\;$or −0.011 (low, medium, or high self-interactions, respectively), $x_{\text{1}}(\text{0})=\text{0}.\text{3}$, $x_{\text{2}}(\text{0})=\text{0}.\text{5}$, $x_{\text{3}}(\text{0})=\text{0}.\text{2}$, $N_{sum}(\text{0})=\text{1}\text{0}\text{0}$). We set $N_{sum\_intial\_guess}=\text{2}\text{0}\text{0}$.

**Table 4:** Average cosine similarity of interaction coefficients (*b*_ij_) where i≠j under varying time intervals (Δt) and noise levels for different methods, using simulated datasets that reflect a stabilizing system of three species.

Time segment		iLV	cLV	gLV_relative	Pearson’s r	Spearman’s r	gLV_absolute
**No noise**	Δt = 1	**1**	0.856	0.743	0.0963	0.0965	0.903
	Δt = 0.5	**1**	0.921	0.840	0.0923	0.100	0.971
	Δt = 0.1	**1**	0.955	0.892	0.0880	0.0901	0.998
**Noise level 0.05**	Δt = 1	**0.979** **(0.0179)**	0.852 (0.00911)	0.738 (0.0135)	0.0969 (0.00582)	0.0994 (0.0134)	0.900 (0.0165)
	Δt = 0.5	**0.990** **(0.00911)**	0.918 (0.00619)	0.835 (0.00783)	0.0914 (0.00365)	0.0969 (0.00495)	0.971 (0.0109)
	Δt = 0.1	0.996 (0.00565)	0.947 (0.00352)	0.871 (0.00814)	0.0884 (0.00160)	0.0900 (0.00217)	0.994 (0.00413)
**Noise level 0.10**	Δt = 1	0.838 (0.159)	0.844 (0.0159)	0.725 (0.0209)	0.0963 (0.0122)	0.102 (0.0167)	**0.875** **(0.0350)**
	Δt = 0.5	0.890 (0.170)	0.904 (0.0121)	0.814 (0.0260)	0.0934 (0.00601)	0.0952 (0.0101)	**0.953** **(0.0356)**
	Δt = 0.1	0.960 (0.0852)	0.851 (0.0337)	0.744 (0.0349)	0.0875 (0.00353)	0.0890 (0.00388)	**0.974** **(0.0216)**

For each noisy group, 20 randomly noisy datasets of relative abundance were generated, and the standard deviation was reported in parentheses following the mean cosine similarities of *b*_ij_. Best performances (Friedman Test, followed by post-hoc one-sided Wilcoxon signed-rank test, with p value less than 0.05) are highlighted in bold. The data were simulated under the second parameter setting ($r_{\text{1}}=\text{0}.\text{2}\text{1}$, $r_{\text{2}}=-\text{0}.\text{4}$, $r_{\text{3}}=\text{0}.\text{1}\text{9}$, $b_{\text{1}\text{1}}=\;b_{\text{2}\text{2}}=b_{\text{3}\text{3}}=\;$0, $b_{\text{1}\text{2}}=-\text{0}.\text{0}\text{2}$, $b_{\text{1}\text{3}}=\text{0}.\text{016}$, $b_{\text{2}\text{1}}=\text{0}.\text{01}$, $b_{\text{2}\text{3}}=-\text{0}.\text{014}$, $b_{\text{3}\text{1}}=-\text{0}.\text{017}$, $b_{\text{3}\text{2}}=\text{0}.\text{02}$, $x_{\text{1}}(\text{0})=\text{0}.\text{3}$, $x_{\text{2}}(\text{0})=\text{0}.\text{5}$, $x_{\text{3}}(\text{0})=\text{0}.\text{2}$, $N_{sum}(\text{0})=\text{1}\text{0}\text{0}$). We set $N_{sum\_intial\_guess}=\text{2}\text{0}\text{0}$.

**Table 5:** Average cosine similarity of interaction coefficients (*b*_ij_) where i≠j under varying time ranges (t) and noise levels for different methods, using simulated datasets that reflect a stabilizing system of three species.

Time range		iLV	cLV	gLV_relative	Pearson’s r	Spearman’s r	gLV_absolute
**No noise**	t = 20	**1**	0.832	0.716	0.148	0.166	0.988
	t = 15	**1**	0.853	0.738	0.102	0.106	0.977
	t = 10	**1**	0.856	0.743	0.0963	0.0965	0.903
**Noise level 0.05**	t = 20	0.875 (0.276)	0.828 (0.00897)	0.711 (0.0102)	0.148 (0.00269)	0.167 (0.00294)	**0.986** **(0.00233)**
	t = 15	**0.993** **(0.00688)**	0.848 (0.00893)	0.731 (0.0106)	0.101 (0.00470)	0.106 (0.00886)	0.974 (0.00537)
	t = 10	**0.979** **(0.0179)**	0.852 (0.00911)	0.738 (0.0135)	0.0969 (0.00582)	0.0994 (0.0134)	0.900 (0.0165)
**Noise level 0.10**	t = 20	0.868 (0.336)	0.819 (0.0129)	0.692 (0.0153)	0.146 (0.00447)	0.164 (0.00416)	**0.981** **(0.00768)**
	t = 15	0.951 (0.144)	0.835 (0.0196)	0.716 (0.0250)	0.101 (0.0110)	0.108 (0.0123)	**0.969** **(0.0136)**
	t = 10	0.838 (0.159)	0.844 (0.0159)	0.725 (0.0209)	0.0963 (0.0122)	0.102 (0.0167)	**0.875** **(0.0350)**

For each noisy group, 20 randomly noisy datasets of relative abundance were generated, and the standard deviation was reported in parentheses following the mean cosine similarities of *b*_ij_. Best performances (Friedman Test, followed by post-hoc one-sided Wilcoxon signed-rank test, with p value less than 0.05) are highlighted in bold. The data were simulated under the second parameter setting ($r_{\text{1}}=\text{0}.\text{2}\text{1}$, $r_{\text{2}}=-\text{0}.\text{4}$, $r_{\text{3}}=\text{0}.\text{1}\text{9}$, $b_{\text{1}\text{1}}=\;b_{\text{2}\text{2}}=b_{\text{3}\text{3}}=\;$0, $b_{\text{1}\text{2}}=-\text{0}.\text{0}\text{2}$, $b_{\text{1}\text{3}}=\text{0}.\text{016}$, $b_{\text{2}\text{1}}=\text{0}.\text{01}$, $b_{\text{2}\text{3}}=-\text{0}.\text{014}$, $b_{\text{3}\text{1}}=-\text{0}.\text{017}$, $b_{\text{3}\text{2}}=\text{0}.\text{02}$, $x_{\text{1}}(\text{0})=\text{0}.\text{3}$, $x_{\text{2}}(\text{0})=\text{0}.\text{5}$, $x_{\text{3}}(\text{0})=\text{0}.\text{2}$, $N_{sum}(\text{0})=\text{1}\text{0}\text{0}$). We set $N_{sum\_intial\_guess}=\text{2}\text{0}\text{0}$.

We also added different levels of self-interaction terms $b_{ii}\;$in simulated datasets. Positive self-interactions are rarely biologically meaningful, which usually lead to unstable dynamics [16,17]. Therefore, $b_{ii}$ was randomly assigned from three negative ranges representing different magnitudes of self-regulations: -0.005 to 0 (low), -0.01 to -0.005 (medium), and -0.015 to -0.01 (high). The metric of cosine similarity remains the same as in Tables 1, 2, 4, and 5, and results are presented in Tables 3 and 6 for periodic-oscillation and stabilizing systems, respectively. We incorporated self-interaction terms ($b_{ii}$) into gLV_absolute modeling for results presented in Tables 3 and 6. However, this was not feasible for iLV, cLV, or gLV_relative due to the identifiability issue mentioned in the Methods section. While gLV_absolute generally achieved the best performance using absolute-abundance inputs, iLV consistently outperformed cLV and gLV_relative using relative-abundance inputs. Under all levels of self-interactions, iLV exhibited stable and reliable performance in capturing the relative interaction strengths among species, with cosine similarity consistently exceeding 0.7. By comparison, both cLV and gLV_relative yielded markedly lower cosine similarities (Tables 3 and 6).

**Table 6:** The cosine similarity of interaction coefficients (*b*_ij_) where i≠j under varying time intervals (Δt) and self-interaction levels for different methods, using simulated datasets that reflect stabilizing systems of three species.

Time segment		iLV	cLV	gLV_relative	Pearson’s r	Spearman’s r	gLV_absolute
**Low self-interaction**	Δt = 1	**0.987**	0.827	0.704	0.139	0.133	0.965
	Δt = 0.5	0.988	0.873	0.787	0.137	0.115	**0.991**
	Δt = 0.1	0.988	0.895	0.843	0.136	0.110	**0.999**
**Medium self-interaction**	Δt = 1	0.812	0.727	0.567	0.164	0.176	**0.973**
	Δt = 0.5	0.887	0.767	0.619	0.164	0.174	**0.993**
	Δt = 0.1	0.819	0.790	0.656	0.165	0.174	**0.999**
**High self-interaction**	Δt = 1	0.874	0.633	0.448	0.169	0.178	**0.970**
	Δt = 0.5	0.874	0.671	0.484	0.169	0.176	**0.992**
	Δt = 0.1	0.876	0.695	0.510	0.169	0.175	**0.999**

Best performances are highlighted in bold. The data were simulated under the first parameter setting ($r_{\text{1}}=\text{0}.\text{2}\text{1}$, $r_{\text{2}}=-\text{0}.\text{4}$, $r_{\text{3}}=\text{0}.\text{1}\text{9}$, $b_{\text{1}\text{1}}=-\text{0}.\text{0}\text{0}\text{1}\text{1},\;-\text{0}.\text{0}\text{0}\text{6}\text{4},\;$or −0.01 (low, medium, or high self-interactions, respectively), $b_{\text{1}\text{2}}=-\text{0}.\text{0}\text{2}$, $b_{\text{1}\text{3}}=\text{0}.\text{016}$, $b_{\text{2}\text{1}}=\text{0}.\text{01}$, $b_{\text{2}\text{2}}=-\text{0}.\text{0}\text{0}\text{2}\text{1},\;-\text{0}.\text{0}\text{0}\text{8}\text{6},\;$or −0.013 (low, medium, or high self-interactions, respectively), $b_{\text{2}\text{3}}=-\text{0}.\text{014}$, $b_{\text{3}\text{1}}=-\text{0}.\text{017}$, $b_{\text{3}\text{2}}=\text{0}.\text{02}$, $b_{\text{3}\text{3}}=-\text{0}.\text{0}\text{0}\text{3}\text{5},\;-\text{0}.\text{0}\text{0}\text{5}\text{3},\;$or −0.011 (low, medium, or high self-interactions, respectively), $x_{\text{1}}(\text{0})=\text{0}.\text{3}$, $x_{\text{2}}(\text{0})=\text{0}.\text{5}$, $x_{\text{3}}(\text{0})=\text{0}.\text{2}$, $N_{sum}(\text{0})=\text{1}\text{0}\text{0}$). We set $N_{sum\_intial\_guess}=\text{2}\text{0}\text{0}$.

Across all scenarios, iLV performed well against competing methods in recovering interaction coefficients (*b*_ij_). In noise-free conditions, iLV achieved perfect or near-perfect cosine similarities between predicted and ground-truth coefficients, demonstrating its accuracy and reliability. While gLV-relative and cLV methods are limited by their reliance on linear approximations and the assumption that $N_{sum}$ stays constant, iLV leverages iterative optimization to refine parameter estimates, resulting in significantly improved accuracy under most of the scenarios. Moreover, the Pearson and Spearman coefficients are generally very small in the four tables, indicating that they cannot be used to measure the interaction strength between different species.

The benchmarking results revealed that iLV’s performance is sensitive to temporal resolution, with smaller time intervals (Δt) yielding more accurate estimates. Longer temporal ranges had similar effects on iLV performance but with some exceptions (such as the second parameter setting when t = 20). In contrast, the performance of cLV and gLV-relative methods plateaued or declined under these conditions, underscoring iLV’s ability to exploit temporal information effectively. As noise levels increased, iLV showed higher variance compared to other simple linear approximation methods, since higher noise levels can make the non-linear optimization process harder by further complicating the non-convex objective function.

When comparing methods across all benchmark scenarios, gLV-absolute performed well with absolute abundance data, but its reliance on such input limits its applicability to microbiome studies, where relative abundance is often the only available measure. In contrast, iLV provides comparable accuracy while using relative abundance, demonstrating its versatility and practical relevance.

### The snowshoe hare-Canadian lynx population dynamics and the *Stylonychia pustula-P. caudatum* mixed culture

In the northern boreal forests of North America, the snowshoe hare (Lepus americanus) serves as the primary food source for the Canadian lynx (Lynx canadensis). When hare populations are abundant, lynx consume approximately two hares every three days, primarily excluding other prey from their diet. As a result, the population dynamics of these two species are tightly interconnected. For over a century, it has been observed that their populations underwent significant fluctuations in cycles lasting approximately 8–11 years [14].

**Fig 3:**
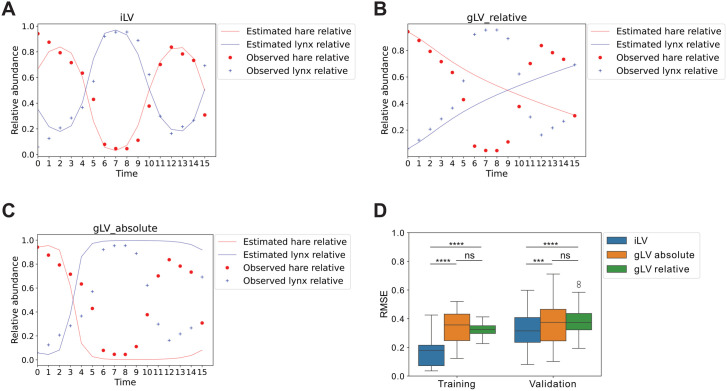
Comparison of predicted and observed relative abundance trajectories for snowshoe hare-Canadian lynx. We set ${𝐍}_{sum\_intial\_guess}=\text{1}\text{0}$ according to Table 7. Panels **A**, **B**, and **C** show the trajectories generated by iLV, gLV_relative, and gLV_absolute models using all 16 pairs of data points, respectively, with observed data overlaid for comparison. iLV aligns closely with observed trajectories (RMSE = 0.109), whereas gLV_relative (RMSE = 0.318) and gLV_absolute (RMSE = 0.425) display higher errors. Panel **D** illustrates the training and validation RMSE distribution across 100 runs, showing iLV significantly outperforming the other methods in training and validation accuracies. Significance is computed relative using the Friedman Test followed by a post-hoc one-sided Wilcoxon signed-rank test (****: p <$10^{-\text{4}}$; ***:p < 0.001; **: p < 0.01; *: p < 0.05; ns: not significant).

We utilized the absolute abundance data of lynx and hare from 1920 to 1935 [18], converting it into relative abundances. To analyze the data, we applied the iLV and gLV_relative models to the relative abundance dataset and gLV_absolute model to the absolute abundance data. The resulting trajectory RMSEs were 0.109, 0.318, and 0.425, respectively, indicating that iLV achieved the lowest RMSE and provided the best agreement between estimated and observed trajectories, as shown in Fig 3A, 3B, and 3C.

**Table 7:** Runtime and sensitivity to $N_{sum\_intial\_guess}$ of the iLV Algorithm using real datasets.

Number of species	Number of time points	$N_{sum\_intial\_guess}$	Runtime (Seconds)	Trajectory RMSE
Lynx-hare				
2	16	0.001	2.11	0.118
2	16	0.01	2.01	0.122
2	16	0.1	2.12	0.114
2	16	1	2.38	0.110
**2**	**16**	**10**	**2.37**	**0.109**
2	16	100	2.40	0.112
2	16	1000	2.22	0.112
*Stylonychia pustula-P. caudatum*				
2	19	0.001	1.95	0.0685
2	19	0.01	2.09	0.0673
2	19	0.1	2.10	0.0673
2	19	1	1.91	0.0684
2	19	10	2.10	0.0673
**2**	**19**	**100**	**1.85**	**0.0673**
2	19	1000	1.89	0.0673
Cheese				
**5**	**11**	**0.001**	**4.29**	**0.149**
5	11	0.01	10.50	0.186
5	11	0.1	4.58	0.173
5	11	1	5.47	0.152
5	11	10	6.91	0.175
5	11	100	4.85	0.186
5	11	1000	3.69	0.186

We tested runtime and sensitivity to $N_{sum\_intial\_guess}$ for all three real datasets used in this paper, which are the lynx-hare dataset, the *Stylonychia pustula-P. caudatum* dataset, and the cheese microbial dataset. The runtime was measured for a single run on a personal laptop, and the number of loops M in Subroutine 1 of the iLV Algorithm was set to 100. We set $N_{sum\_intial\_guess}$ to 10, 100 and 0.001 for lynx-hare, *Stylonychia pustula-P. caudatuma* and cheese microbial datasets, respectively in our data analysis. The rows corresponding to the lowest trajectory RMSE are in bold.

To further evaluate model performance, we randomly split the relative abundance dataset into 10 pairs (hare and lynx) of data points for parameter training and 6 pairs of data points for validation across 100 runs. The performance of iLV, gLV_relative, and gLV_absolute was compared using training and validation trajectory RMSE as shown in Fig 3D. The cLV model was excluded from this analysis, as it can only estimate $r_{i}-r_{D}$ and $b_{ij}-b_{Dj}$, where species D has the lowest observed variance of relative abundance among all species. Without explicit estimates for $r_{i}$ and $b_{ij}$, the trajectories of relative abundances cannot be recovered, making trajectory RMSE computation infeasible for cLV.

The iLV model significantly outperformed gLV_relative and gLV_absolute in training RMSE, with p-values $\text{1}.\text{7}\text{2}\times {\text{1}\text{0}}^{-\text{1}\text{7}}$ and $\text{8}.\text{9}\text{3}\times {\text{1}\text{0}}^{-\text{1}\text{5}}$, respectively (Friedman Test followed by a post-hoc one-sided Wilcoxon signed-rank test). No significant difference in training RMSE was observed between gLV_relative and gLV_absolute. For validation RMSE, iLV significantly outperformed gLV_relative and gLV_absolute, with p-values of$\;\text{2}.\text{3}\text{2}\times {\text{1}\text{0}}^{-\text{5}}$ and $\text{3}.\text{8}\text{1}\times {\text{1}\text{0}}^{-\text{4}}$, respectively. No significant difference in validation RMSE was detected between gLV_relative and gLV_absolute. Overall, the iLV model consistently performed best among the three methods during training and validation, which underscores iLV’s reliability.

**Fig 4:**
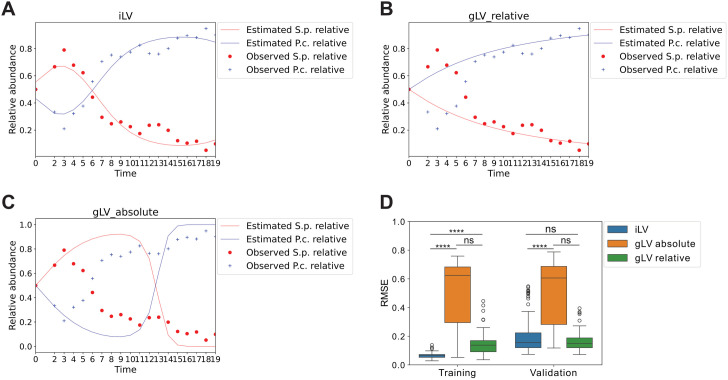
Comparison of predicted and observed relative abundance trajectories for *Stylonychia pustulata* and *P. caudatum.* We set ${𝐍}_{sum\_intial\_guess}=\text{1}\text{0}\text{0}$ according to Table 7. Panels **A**, **B**, and **C** show the trajectories generated by iLV, gLV_relative, and gLV_absolute models using all 19 pairs of data points, respectively, with observed data overlaid for comparison. iLV aligns closely with observed trajectories (RMSE = 0.0673), whereas gLV_relative (RMSE = 0.163) and gLV_absolute (RMSE = 0.383) display higher errors. Panel **D** illustrates the training and validation RMSE distribution across 100 runs, showing iLV significantly outperforming the other methods in training accuracy and maintainin significantly better validation performance than gLV_absolute. Significance is computed relative using the Friedman Test followed by a post-hoc one-sided Wilcoxon signed-rank test (****: p <$10^{-\text{4}}$; ***:p < 0.001; **: p < 0.01; *: p < 0.05; ns: not significant).

A dataset containing the abundances of *Stylonychia pustulata* grown in mixture with *P. caudatum* on Osterhout medium was recorded in *The Struggle for Existence* [19] and digitized by Mühlbauer et al. [20]. Absolute abundances of these two species were recorded in a 25-day period, and we converted them to relative abundances. Day 2 was skipped, while individual values were missing and only the mean was reported for days 20–25 in the original data. Besides, the system started to plateau after day 19, so we modeled mean abundances (of three replicates) before day 19. The fitted results are given in Fig 4. We compared the performances of iLV, gLV_relative, and gLV_absolute, and iLV had the lowest RMSE (0.0673) compared to the other two models (with RMSE of 0.163 and 0.383, respectively). iLV also captured the system oscillation between day 0 and day 7, while the other two models could not.

Similarly, we randomly split the whole dataset into 12 pairs of data points for training and 7 pairs of data points for validation across 100 runs, and the results are shown in Fig 4D. iLV performed significantly better than gLV_relative and gLV_absolute in training RMSE. As for validation, iLV performed significantly better than gLV_absolute, but there was no significant difference between iLV and gLV_relative in validation RMSE.

### A cheese microbial community

Mounier et al. previously quantified cell counts of five microbial groups within a cheese microbial community over a 21-day period [15]. We converted these absolute counts to relative abundances for modeling purposes. These groups include *D. hansenii*, *Y. lipolytica*, *G. candidum*, *Leucobacter sp.*, and a bacterial group composed of *Arthrobacter arilaitensis*, *Hafnia alvei*, *Corynebacterium casei*, *Brevibacterium aurantiacum*, and *Staphylococcus xylosus*. The system plateaued around day 10, and we modeled the dynamic phase between days 0 and 10. We applied our iLV model to this dataset with $N_{sum\_intial\_guess}$ set to 0.001 (see Table 7), and the trajectory RMSE is 0.149. For comparison, we also applied the gLV_relative and gLV_absolute models, which yielded trajectory RMSEs of 0.327 and 0.296, respectively.

**Fig 5:**
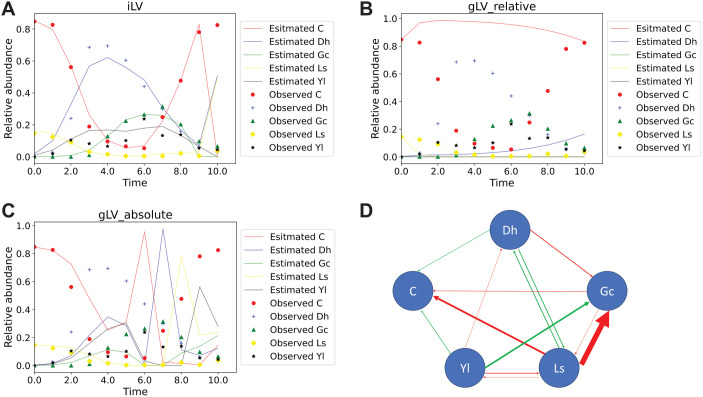
Predicted abundance trajectory and interactions of the cheese microbial community. We set ${𝐍}_{sum\_intial\_guess}=\text{0}.\text{0}\text{0}\text{1}$ according to Table 7. Panels **A**, **B**, and **C** show the trajectories generated by iLV, gLV_relative, and gLV_absolute models, respectively, with observed data overlaid for comparison. iLV aligns closely with observed trajectories (RMSE = 0.149), whereas gLV_relative (RMSE = 0.327) and gLV_absolute (RMSE = 0.296) display higher errors. Panel **D** shows the predicted interaction network. The green arrows mean promotion, and the red arrows mean inhibition. The thickness of the arrows is proportional to the effect size of promotion or inhibition, indicated by the magnitude of b_ij_. Only interactions that are at least 50 times larger than the minimum predicted interaction were plotted. Dh is *D. hansenii*, Yl is *Y. lipolytica*, Gc is *G. candidum*, Ls is *Leucobacter sp.*, and C is a bacterial group composed of *Arthrobacter arilaitensis*, *Hafnia alvei*, *Corynebacterium casei*, *Brevibacterium aurantiacum*, and *Staphylococcus xylosus*.

Significant interactions among the microbial species, as predicted by the iLV model, are shown in Fig 5D. Some of these interactions are supported by previous studies [15]. For example, *Y. lipolytica* has been shown to reduce the viability of *D. hansenii* during the stationary phase [15]. However, not all predicted interactions align with prior findings. For instance, *Y. lipolytica* was reported to inhibit the mycelial growth of *G. candidum*, rather than promoting it. This inhibition disrupted the typical mold-like mycelial structure of *G. candidum*, leading to the formation of spaghetti-like morphologies [15]. It is important to note, however, that Mounier et al. investigated pairwise interactions through two-species co-culture experiments [15], which may not fully capture the complexity of interactions within a multispecies community.

### Runtime and sensitivity to $N_{sum\_intial\_guess}$ of the iLV Algorithm

We tested the runtime of the iLV Algorithm in both real and simulated datasets under different values of $N_{sum\_intial\_guess}$ (varying from 0.001 to 1000), and the results are shown in Tables 5 and 6, respectively. In the lynx-hare dataset, trajectory RMSE had minor fluctuations around 0.11 and achieved the minimum of 0.109 when $N_{sum\_intial\_guess}=\text{1}\text{0}$. Similarly, trajectory RMSE stayed rather stable around 0.068 and achieved the minimum of 0.0673 when $N_{sum\_intial\_guess}=\text{1}\text{0}\text{0}$ in the *Stylonychia pustula-P. caudatum* dataset. The cheese microbial dataset exhibited slightly higher fluctuations and achieved the minimum RMSE of 0.149 when $N_{sum\_intial\_guess}=\text{0}.\text{0}\text{0}\text{1}$. For the simulated datasets, generated using the two parameter settings applied in benchmarking with 10% Gaussian noise added, the trajectory RMSE remained consistent across different values of $N_{sum\_intial\_guess}$. Based on these results, we adopted $N_{sum\_intial\_guess}=\text{2}\text{0}\text{0}$ in benchmarking analyses without loss of generality. Overall, the trajectory RMSE is not highly sensitive to the value of $N_{sum\_intial\_guess}$ for small systems, but we noticed a small increase in sensitivity as the system becomes larger and more complicated.

The iLV model’s computational bottleneck lies in the nonlinear optimization step (Subroutine 2), which uses leastsq() and least_squares() functions from the SciPy library [21]. The time complexities of the Levenberg–Marquardt algorithm (implemented in both leastsq() and least_squares(method = ’lm’)) and the Trust Region Reflective algorithm (least_squares(method = ’trf’)), both scale approximately with *O*($k*(n^{\text{2}}m+n^{\text{3}})$) when the Jacobian of cost function F(x) is analytically solvable, where *n* is the number of parameters, *m* is the number of timepoints, and *k* is the number of iterations until convergence (depending on the problem, starting point, etc.) [22,23]. In our experiments, the runtime was acceptable for low-dimensional systems (see Tables 5 and 6). It generally increases with the number of species and the number of time points, while $N_{sum\_intial\_guess}$ can also affect runtime by providing different starting points and thus affecting the number of iterations required for convergence.

## Discussion

This study presents the iterative Lotka-Volterra (iLV) model as a novel approach for parameter estimation using relative abundance data, which integrates iterative refinement with non-linear optimization. The iLV model outperformed existing methodologies, such as the compositional Lotka-Volterra (cLV) model and generalized Lotka-Volterra (gLV_relative) approaches, in recovering interaction coefficients of simulation data under various noise levels and temporal resolutions. Moreover, compared with cLV and gLV_relative, iLV demonstrated more stable and reliable performance in capturing the relative interaction strengths across species under different magnitudes of self-interactions.

Real-world applications, such as Canadian lynx-snowshoe hare, *Stylonychia pustula*-*P. caudatum*, and the cheese microbial community datasets, further validated the practicality of iLV. In these cases, iLV successfully reconstructed system trajectory with low RMSE values, demonstrating its ability to handle compositional data without sacrificing model interpretability. For instance, in the lynx-hare system, iLV accurately captured population dynamics, aligning closely with observed data (RMSE = 0.109). The iterative design of iLV is a significant improvement over traditional linear regression methods. By iteratively solving ordinary differential equations and refining parameter estimates, iLV minimizes the root mean square error (RMSE) between observed and predicted relative abundances.

Despite its effectiveness, the iLV model’s computational bottleneck lies in the nonlinear optimization step (Subroutine 2), which uses leastsq() and least_squares() functions from the SciPy library [21]. These functions can be computationally intensive, particularly for systems with many species or dense time-series data. In our experiments, the runtime was acceptable for low-dimensional systems (see Tables 5 and 6), but challenges will occur for higher-dimensional systems. To address scalability concerns, several strategies can be considered in future work. One is the parallelization of key components, such as distributed optimization across multiple initializations [24]. Furthermore, using sparse Jacobians or approximate Hessians could potentially reduce computation without a major loss in accuracy, as has been shown in large-scale numerical optimization problems [25,26]. These approaches would enhance the scalability of iLV and facilitate its application to larger and more complex microbiome datasets, where compositional data with many taxa and high temporal resolution are increasingly common [10,11]. Another solution would be dimensionality reduction. For example, Phuongan et al. reduced more than 11,000 microbial Operational Taxonomic Units (OTUs) into 14 subgroups by clustering their blooming times, and they modeled on the subgroups instead of OTUs [27].

Besides, incorporating additional ecological constraints, such as carrying capacities or environmental perturbations, could improve the realism of the model. Moreover, an initial guess of the inter-species sum of absolute abundances $N_{sum\_intial\_guess}$ is needed for iLV, and users may tweak it several times for better prediction accuracy. Sensitivity of $N_{sum\_intial\_guess}$ is dataset-specific, and datasets involving larger and more complicated systems are more likely to be sensitive to $N_{sum\_intial\_guess}$ in our experiments (see Tables 7 and 8). The number of data points also matters for iLV performance, and data interpolation may help with inadequate input data while also introducing additional errors.

**Table 8:** Runtime and sensitivity to $N_{sum\_intial\_guess}$ of the iLV Algorithm using simulated datasets.

Number of species	Number of time points	$N_{sum\_intial\_guess}$	Runtime (Seconds)	Trajectory RMSE
Periodic oscillation				
3	11	0.001	2.56	0.0156
3	11	0.01	4.07	0.0156
3	11	0.1	4.30	0.0156
3	11	1	3.56	0.0156
3	11	10	3.66	0.0156
3	11	100	3.94	0.0156
**3**	**11**	**200**	**4.06**	**0.0156**
3	11	1000	3.55	0.0156
Stabilizing system				
3	11	0.001	2.16	0.0102
3	11	0.01	2.46	0.0102
3	11	0.1	2.29	0.0102
3	11	1	2.24	0.0102
3	11	10	2.13	0.0102
3	11	100	10.59	0.0102
**3**	**11**	**200**	**3.54**	**0.0102**
3	11	1000	2.31	0.0102

We utilized two parameter settings from benchmarking. The first parameter setting represents **periodic oscillations** among three species: $r_{\text{1}}=\text{0}.\text{3}\text{1}$, $r_{\text{2}}=-\text{0}.\text{6}$, $r_{\text{3}}=\text{0}.\text{2}\text{9}$, $b_{\text{1}\text{2}}=-\text{0}.\text{0}\text{1}$, $b_{\text{1}\text{3}}=\text{0}.\text{011}$, $b_{\text{2}\text{1}}=\text{0}.\text{009}$, $b_{\text{2}\text{3}}=-\text{0}.\text{01}$, $b_{\text{3}\text{1}}=-\text{0}.\text{012}$, $b_{\text{3}\text{2}}=\text{0}.\text{015}$, $x_{\text{1}}(\text{0})=\text{0}.\text{3}$, $x_{\text{2}}(\text{0})=\text{0}.\text{5}$, $x_{\text{3}}(\text{0})=\text{0}.\text{2}$, $N_{sum}(\text{0})=\text{1}\text{0}\text{0}$. The second parameter setting reflects a **stabilizing system** of three species: $r_{\text{1}}=\text{0}.\text{2}\text{1}$, $r_{\text{2}}=\text{0}.\text{4}$, $r_{\text{3}}=\text{0}.\text{1}\text{9}$, $b_{\text{1}\text{2}}=\text{0}.\text{0}\text{2}$, $b_{\text{1}\text{3}}=\text{0}.\text{016}$, $b_{\text{2}\text{1}}=\text{0}.\text{01}$, $b_{\text{2}\text{3}}=-\text{0}.\text{014}$, $b_{\text{3}\text{1}}=-\text{0}.\text{017}$, $b_{\text{3}\text{2}}=\text{0}.\text{02}$, $x_{\text{1}}(\text{0})=\text{0}.\text{3}$, $x_{\text{2}}(\text{0})=\text{0}.\text{5}$, $x_{\text{3}}(\text{0})=\text{0}.\text{2}$, $N_{sum}(\text{0})=\text{1}\text{0}\text{0}.$ The simulated time ranges were 0–10 with step size 1, and 10% random Gaussian noise was added. The runtime was measured for a single run on a personal laptop, and the number of loops M in Subroutine 1 of the iLV Algorithm was set to 100. Both simulation parameter settings were insensitive to $N_{sum\_intial\_guess}$, and we used $N_{sum\_intial\_guess}=\text{2}\text{0}\text{0}$ in all benchmarking without loss of generality that is highlighted in bold.

In summary, the iterative Lotka-Volterra model offers a robust framework for analyzing compositional data. By addressing limitations in existing methodologies and demonstrating superior performance across simulated and real-world datasets, iLV sets a new approach for species interaction modeling. Future efforts to expand data dimensionality and ecological assumptions (such as perturbations) will further cement its utility in ecological and microbiome research.

## Methods

### The generalized Lotka-Volterra model, defined with relative abundances

The Lotka-Volterra model was first proposed by Alfred James Lotka in 1910 for chemical reactions [28], and in 1925 he used the model to study predator-prey interactions [4]. In 1926, the same model was published by Vito Volterra [29]. While the Lotka-Volterra model only depicts systems of two species, the generalized Lotka-Volterra (gLV) was proposed to model systems of more than two species. The gLV model is widely used in describing the dynamics of a set of organisms, which takes the form of (where *N*_1_, …, *N*_m_ are absolute abundances of the different organisms in the system, and *r*_i_ and *b*_ij_ are coefficients):


$$\left\{ \begin{array}{c} \frac{dN_{\text{1}}}{dt}=r_{\text{1}}N_{\text{1}}+\sum_{j=\text{1}}^{m}b_{\text{1}j}N_{\text{1}}N_{j}\\ \frac{dN_{\text{2}}}{dt}=r_{\text{2}}N_{\text{2}}+\;\sum_{j=\text{1}}^{m}b_{\text{2}j}N_{\text{2}}N_{j}\\ \ldots \ldots \\ \frac{dN_{m}}{dt}=r_{m}N_{m}+\sum_{j=\text{1}}^{m}b_{mj}N_{m}N_{j} \end{array}\right.\;$$ (1)

Relative abundances (*x*_1_, …, *x*_m_) and absolute abundances can be converted mutually if the sum of absolute abundances of all species in the system is given:


$$N_{sum}=\sum_{i=\text{1}}^{m}N_{i}$$ (2)

$$x_{i}=\frac{N_{i}}{N_{sum}}$$ (3)

According to equations (1)(2)(3), the gLV model can be rewritten with *x*_1_, …, *x*_m_ and *N*_*sum*_:


$$\left\{ \begin{array}{c} \frac{dN_{sum}}{dt}=\;\sum_{i=\text{1}}^{m}{(r}_{i}x_{i}N_{sum}+\sum_{j=\text{1}}^{m}b_{ij}x_{i}x_{j}N_{sum}^{\text{2}})\\ \frac{dx_{\text{1}}}{dt}={(r}_{\text{1}}x_{\text{1}}N_{sum}+\sum_{j=\text{1}}^{m}b_{\text{1}j}x_{\text{1}}x_{j}N_{sum}^{\text{2}}-x_{\text{1}}\frac{dN_{sum}}{dt})/N_{sum}\\ \frac{dx_{\text{2}}}{dt}={(r}_{\text{2}}x_{\text{2}}N_{sum}+\sum_{j=\text{1}}^{m}b_{\text{2}j}x_{\text{2}}x_{j}N_{sum}^{\text{2}}-x_{\text{2}}\frac{dN_{sum}}{dt})/N_{sum}\\ \ldots \ldots \\ \frac{dx_{m}}{dt}={(r}_{m}x_{m}N_{sum}+\sum_{j=\text{1}}^{m}b_{mj}x_{m}x_{j}N_{sum}^{\text{2}}-x_{m}\frac{dN_{sum}}{dt})/N_{sum} \end{array}\right.\;$$ (4)

where the first sub-equation in (4) holds as the derivative of the sum equals the sum of derivatives and the rest of equations (4) holds as the following:


$$\frac{dx_{i}}{dt}=\;\frac{d\;(\frac{N_{i}}{N_{sum}})}{dt}=\;\frac{\frac{dN_{i}}{dt}N_{sum}-N_{i}\frac{dN_{sum}}{dt}}{N_{sum}^{\text{2}}}=\;\frac{\frac{dN_{i}}{dt}-x_{i}\frac{dN_{sum}}{dt}}{N_{sum}}=\;\frac{r_{i}N_{i}+\sum_{j=\text{1}}^{m}b_{ij}N_{i}N_{j}-x_{i}\frac{dN_{sum}}{dt}}{N_{sum}}=\;\frac{r_{i}x_{i}N_{sum}+\sum_{j=\text{1}}^{m}b_{ij}x_{i}x_{j}N_{sum}^{\text{2}}-x_{i}\frac{dN_{sum}}{dt}}{N_{sum}}$$

We used odeint() function in scipy package [21] to solve the ordinary differential equation system (4) numerically when the initial values of *x*_1_, …, *x*_m_ and *N*_*sum*_ are given.

### Parameter estimation by linear approximation and regression

Multiple investigators have used the following linear approximations for the generalized Lotka-Volterra model when absolute abundances are given [6,8,15,30]:


$$\frac{\Delta (\text{ln}N_{i})}{\Delta t}\approx \frac{d\left(\text{ln}N_{i}\right)}{dt}=\frac{\frac{dN_{i}}{dt}}{N_{i}}=r_{i}+\sum_{j=\text{1}}^{m}b_{ij}N_{j}$$ (5)

where $\Delta \left(\text{ln}N_{i}\right)=$ $\text{ln}N_{i}\left(t_{k+\text{1}}\right)-\text{ln}N_{i}(t_{k}\backslash )$ is the difference of $\text{ln}N_{i}\;$ at two adjacent time points $t_{k+\text{1}}$ and $t_{k}$, and $\Delta t=\;t_{k+\text{1}}-t_{k}$

By linear approximation (5), $r_{i}$ is the intercept and $b_{ij}$ are slopes of the linear regression where $\frac{\Delta (\text{ln}N_{i})}{\Delta t}$ is the dependent variable and $N_{j}$ are independent variables. When absolute abundance data are available, parameters can be estimated as the least square solutions of such linear regressions. In benchmarking, we referred to such method as gLV_absolute.

Since absolute abundances and relative abundances can be mutually converted when the sum of absolute abundances is known, we were inspired by the linear approximation method and came up with a novel iterative method for estimating parameters using the relative abundance presentation of the generalized Lotka-Volterra model (as indicated by equation system (4)).

### The iterative Lotka-Volterra (iLV) algorithm

The iterative Lotka-Volterra (iLV) algorithm has two subroutines: (1) The iterative subroutine, which utilizes linear optimization, and (2) The least square estimation subroutine, which is a non-linear optimization using the results from the iterative subroutine as the starting point of optimization.

When relative abundances $x_{i\_observed}$ of the *i*-th organism at different time points are given and we have an initial guess $N_{sum\_intial\_guess}\;$ of $N_{sum}$, we propose the following iterative subroutine to improve parameter estimation to minimize the root-mean-square (RMSE) between estimated and observed relative abundances:

**Table:** 

Subroutine 1: the iterative subroutine.
**Step 1:** $N_{i}^{\text{0}}=x_{i\_observed}N_{sum\_intial\_guess}.$ Then, use linear approximation (5) and calculate the least square solution of linear regressions. Denote the solution as $r_{i}^{\text{0}}$, $b_{ij}^{\text{0}}$. **Step 2:** For *k* from 0 to *M* (e.g., 99): Solve ordinary differential equation (ODE) system (4) numerically by odeint($r_{i}^{k}$, $b_{ij}^{k}$, $N_{sum\_initial\_guess}$, $x_{i\_observed}(\text{0})$) and calculate RMSE between estimated and observed relative abundances. Please note $x_{i}(\text{0}$are estimated as the observed relative abundances at time point 0, and $N_{sum}(\text{0})$ is estimated as $N_{sum\_intial\_guess}$. Also, denote $N_{sum}^{k+\text{1}}$ as the trajectory of the sum of absolute abundances returned by the odeint() function. Let $N_{i}^{k+\text{1}}=x_{i\_observed}N_{sum}^{k+\text{1}}$. Then, use linear approximation (5) and calculate the least square solution of linear regressions. Denote the solution as $r_{i}^{k+\text{1}}$, $b_{ij}^{k+\text{1}}$. Return $r_{i}^{k}\;$ and $b_{ij}^{k}$ with the minimal RMSE between estimated and observed relative abundances. $x_{i}(\text{0})$ are estimated as the observed relative abundances at time point 0, and $N_{sum}(\text{0})$ is estimated as $N_{sum\_intial\_guess}$.

The motivations behind the iterative algorithm can be described as follows. The first step gives an initial estimate of the parameters ($r_{i}$, $b_{ij})$ assuming constant total absolute abundance across time. The assumption of constant total absolute abundance across time is not correct in most situations and the total absolute abundance calculated based on the estimated parameters using the gLV model may be closer to the real values. Therefore, we re-estimate the parameters using the estimated total absolute abundance together with the observed relative abundance, resulting in a more accurate estimation of absolute abundances. We iterate this process multiple times and choose the parameters as the set having the lowest RMSE between the theoretical relative abundance and observed relative abundance.

Note that if we multiply $N_{sum\_intial\_guess}$ by a constant such that $N_{sum\_initial\_guess}^{\left(c\right)}={c\;N}_{sum\_intial\_guess}$, the resulting $r_{i}$ estimate will not change, while the resulting interaction parameters will be changed to $b_{ij}/c$ from equation (5) in the first step. Therefore, the estimated interaction coefficients will differ by a constant which will not impact the relative abundance across time [12].

We note that the iterative subroutine was based on intuition and intended to obtain an initial estimate of the parameters ($r_{i}$, $b_{ij})$, which can be used as the starting point for the following least square estimation. There is no guarantee that the estimates from the iterative subroutine converge to the real parameter values.

#### Subroutine 2: the least square estimation subroutine.

We used least_squares() and leastsq() functions in scipy package [21] to find a local minimum of the cost function F(x), given initial guesses of the parameters:


$$F(x)=\text{0}.\text{5}\sum_{j=\text{1}}^{m}{f_{j}(x)}^{\text{2}}$$

$$f_{j}(x)=x_{j}-{\left[odeint\left(ODEsystem(\text{4}),\;r_{i},\;b_{ik},\;x_{i}(\text{0}),\;N_{sum}(\text{0})\right)\right]}_{j},\;where\;i,\;k=\text{1},\text{2},..,m$$

Given different initial guesses of the parameters, least_squares() and leastsq() functions may return different estimated parameters corresponding to different local minima of the cost function F(x). Therefore, a close-enough initial guess of parameters is crucial. We used the results returned by Subroutine 1 as the initial guesses for $r_{i}$, $b_{ij}$, observed relative abundances at time point 0 as the initial guesses for $x_{i}(\text{0})$. There are three optimization methods implemented in least_squares() function for finding a nearby local minimum, which are Trust Region Reflective (TRF) algorithm [22], Levenberg-Marquardt (LM) algorithm [23] and dogbox algorithm [31]. The dogbox algorithm is suitable for a small number of variables and may exhibit slow convergence when the rank of Jacobian is less than the number of variables [31], and it had poor performance and stability in our trials, so we only used Trust Region Reflective and Levenberg-Marquardt optimizations. We also included leastsq() function that has a slightly different implementation of Levenberg-Marquardt (LM) algorithm, as compared to least_squares() function. In our data analysis, we compared the trajectory RMSE of least_squares(method = “trf” or method = “lm”) and leastsq(), and returned the results with the lowest trajectory RMSE.

Notably, while the growth coefficients $r_{i}$ in the gLV model are identifiable using relative abundance data, interaction coefficients $b_{ij}$ are only identifiable up to a multiplicative constant [12]. Consequently, the interaction coefficients estimated by the iLV algorithm approximate the true interaction coefficients up to a constant. This property allows us to use the estimated parameters to compare the relative strengths of microbial interactions. Besides, for better performance of the iLV Algorithm, users have to tune $N_{sum\_intial\_guess}$ that returned a local minimum of trajectory RMSE. Sensitivity to $N_{sum\_intial\_guess}$ is dataset-specific, and datasets involving larger and more complicated systems are more likely to be sensitive to $N_{sum\_intial\_guess}$ in our experiments (see Tables 5 and 6). We suggest that investigators carefully compare the trajectories of the relative abundances of the different microbes based on the gLV model under the estimated parameters with the observed relative abundances of the microbes. Their close approximation suggests that the estimated interactions are close to the true underlying interactions.

### Identifiability issue of self-interaction terms

At each time point, only the relative abundances of the species are available, which satisfy the compositional constraint $\sum_{j=\text{1}}^{m}x_{j}=\text{1}$. Thus, the relative abundance of the first species can be represented as $x_{\text{1}}=\text{1}-\sum_{j=\text{2}}^{m}x_{j}$. This expression was substituted into the first step of Subroutine 1 in the iLV algorithm to obtain:


$$\begin{array}{cc} \frac{\Delta \left(\text{ln}({x_{i}N}_{sum\_initial\_guess})\right)}{\Delta t} & =\frac{\Delta \left(\text{ln}x_{i}\right)}{\Delta t}\approx \frac{d\left(\text{ln}x_{i}\right)}{dt}=\frac{\frac{dx_{i}}{dt}}{x_{i}}\\ & =r_{i}+b_{i\text{1}}(\text{1}-\sum_{j=\text{2}}^{m}x_{j})+\sum_{j=\text{2}}^{m}b_{ij}x_{j}=r_{i}+b_{i\text{1}}+\sum_{j=\text{2}}^{m}{(b}_{ij}-b_{i\text{1}})x_{j} \end{array}$$ (6)

In equation (6), while $r_{i}+b_{i\text{1}}$ and $b_{ij}-b_{i\text{1}}$ are identifiable, there are infinitely many solutions for $r_{i}$ and $b_{ij}$. Similar identifiability issues also arise in the cLV and gLV_relative models [13]. To overcome this issue, we assumed self-interaction terms $b_{ii}$ to be 0 when modeling relative abundances with iLV, cLV, or gLV_relative. We also investigated how the presence of self-interactions impacted the estimation of $b_{ij}$ (where i ≠ j) in our simulation studies.

### Evaluation metrics

We used trajectory Root Mean Square Error (RMSE) to measure the performance of our algorithm (where m is the number of microbial species in the system and n + 1 is the number of sampled time points):


$$g_{j}(t_{k})=x_{j}(t_{k})-{\left[odeint\left(ODEsystem(\text{4}),\;r_{i},\;b_{il},\;x_{i}(\text{0}),\;N_{sum}(\text{0})\right)\left(t_{k}\right)\right]}_{j}$$

$$\text{RMSE}=\sqrt{\frac{\sum_{k=\text{0}}^{n}\sum_{j=\text{1}}^{m}g_{j}^{\text{2}}(t_{k})}{(n+\text{1})m}}\;,\;where\;i,\;l=\text{1},\text{2},..,m$$

Cosine similarities of *b*_ij_ between estimated and ground truth values were also used as an evaluation metric in benchmarking. We concatenated the estimated values of all *b*_ij_ (where i = 1,2,..,m, and j ≠ i) as vector A, and we concatenated the ground truth value of all *b*_ij_ vector B. Then, cosine similarity is the cosine of the angle between vector A and vector B.
