# Correction: Quantifying microbial interactions based on compositional data using an iterative approach for solving generalized Lotka-Volterra equations

**DOI:** 10.1371/journal.pcbi.1013876

**Published:** 2026-01-06

**Authors:** Fengzhu Sun, Yue Huang, Tianqi Tang, Xiaowu Dai

After the publication of this article [1] the authors became aware that a closely related study was inadvertently omitted. Specifically, the work by Li et al [2] developed an iterative expectation-maximization (EM) algorithm, BEEM, to estimate parameters in generalized Lotka-Volterra models under the assumption of sparsity in the interaction coefficient matrix. As noted by Li et al. [2], BEEM is not suitable for systems with a small number of species, particularly fewer than six. In contrast, the current version of iLV [1] is designed to work with a relatively small number of species without assuming sparsity in the interaction matrix. Therefore, the application scenarios of BEEM and iLV are complementary, and their performance cannot be directly compared.

The authors apologize for the oversight in not citing Li et al. [2] in our manuscript [1].
