# Correction: Correction: Quantifying microbial interactions based on compositional data using an iterative approach for solving generalized Lotka-Volterra equations

**DOI:** 10.1371/journal.pcbi.1013971

**Published:** 2026-02-13

**Authors:** The PLOS Computational Biology Staff

## Notice of Republication

This article was republished on 29^th^ January 2026 to include references [1] & [2] which were missing from the original correction.

The publisher apologizes for the errors. Please download this article again to view the correct version. The originally published, uncorrected article and the republished, corrected articles are provided here for reference.

## Supporting information

S1 File Originally published, uncorrected article correction. (PDF)

S2 File Republished, corrected article correction. (PDF)
